# Behaviour of Vascular Smooth Muscle Cells on Amine Plasma-Coated Materials with Various Chemical Structures and Morphologies

**DOI:** 10.3390/ijms21249467

**Published:** 2020-12-12

**Authors:** Ivana Nemcakova, Lucie Blahova, Petr Rysanek, Andreu Blanquer, Lucie Bacakova, Lenka Zajíčková

**Affiliations:** 1 Laboratory of Biomaterials and Tissue Engineering, Institute of Physiology of the Czech Academy of Sciences, v.v.i., Videnska 1083, 142 20 Prague 4, Czech Republic; Andreu.BlanquerJerez@fgu.cas.cz (A.B.); lucie.bacakova@fgu.cas.cz (L.B.); 2 Central European Institute of Technology—CEITEC, Masaryk University, Kamenice 5, 625 00 Brno, Czech Republic; lucie.blahova@ceitec.muni.cz (L.B.); lenkaz@physics.muni.cz (L.Z.); 3 Department of Physics, Faculty of Science, University of J. E. Purkyne in Usti nad Labem, Pasteurova 15, 400 96 Usti nad Labem, Czech Republic; petr.rysanek@ujep.cz; 4 Department of Physical Electronics, Faculty of Science, Masaryk University, Kotlarska 2, 611 37 Brno, Czech Republic; 5 Central European Institute of Technology—CEITEC, Brno University of Technology, Purkynova 123, 612 00 Brno, Czech Republic

**Keywords:** amine plasma polymer, bioactive coating, polycaprolactone nanofibers, substrate morphology, cell adhesion, cell proliferation

## Abstract

Amine-coated biodegradable materials based on synthetic polymers have a great potential for tissue remodeling and regeneration because of their excellent processability and bioactivity. In the present study, we have investigated the influence of various chemical compositions of amine plasma polymer (PP) coatings and the influence of the substrate morphology, represented by polystyrene culture dishes and polycaprolactone nanofibers (PCL NFs), on the behavior of vascular smooth muscle cells (VSMCs). Although all amine-PP coatings improved the initial adhesion of VSMCs, 7-day long cultivation revealed a clear preference for the coating containing about 15 at.% of nitrogen (CPA-33). The CPA-33 coating demonstrated the ideal combination of good water stability, a sufficient amine group content, and favorable surface wettability and morphology. The nanostructured morphology of amine-PP-coated PCL NFs successfully slowed the proliferation rate of VSMCs, which is essential in preventing restenosis of vascular replacements in vivo. At the same time, CPA-33-coated PCL NFs supported the continuous proliferation of VSMCs during 7-day long cultivation, with no significant increase in cytokine secretion by RAW 264.7 macrophages. The CPA-33 coating deposited on biodegradable PCL NFs therefore seems to be a promising material for manufacturing small-diameter vascular grafts, which are still lacking on the current market.

## 1. Introduction

Cardiovascular diseases are an increasing population health problem, and are a leading cause of morbidity and mortality. Although pharmacological treatment and minimally invasive surgical techniques offer a growing therapy option, a surgical bypass of blood vessels continues often to be a necessary life-saving procedure. Autologous veins or arteries are still considered to be the gold standard for vascular grafts [1]. However, many patients do not have suitable blood vessels available because of previous surgery or disease. Therefore, the development of tissue-engineered vascular grafts has been rigorously pursued.

Currently used vascular substitutes are manufactured from polymeric materials because of their low cost, easy processing, and good tenability. The most widely used polymers are expanded polytetrafluoroethylene (ePTFE) and polyethylene terephthalate (PET; Dacron) [2,3]. However, these polymers are not biodegradable, and thus they are not able to grow with child patients to remodel or to self-repair in vivo. In addition, ePTFE and PET, although they are in clinical use, are not suitable for manufacturing small-diameter (<6 mm) vascular grafts because of the high rate of restenosis of these grafts. A search for novel better materials, more promising for this application, is therefore highly desirable [4]. Other biodegradable polymers with a tunable degradation rate enabling the gradual removal and replacement of the polymer by newly formed host tissue are therefore currently under investigation [1].

The pristine form of most synthetic polymers has a bio-inert and hydrophobic surface, which often causes problems with the adsorption of proteins in an appropriate spectrum and geometrical conformation, resulting in poor cell adhesion [5,6]. Therefore, various surface modification techniques have been extensively studied to create new hydrophilic, biocompatible, and bioactive materials without affecting the bulk characteristics [7]. Surface amination is an especially attractive approach, because protonated amine groups create electrostatic interactions with negatively charged proteins and cells [8]. Amine groups can be created on the surface of polymers by classical wet chemistry. This leads to a modification with a well-defined chemical structure, depending on the precursor that is used. Nevertheless, the process usually includes several time-consuming and challenging steps [9,10]. Aminolysis reaction often employs toxic reagents such as ethylenediamine [11,12,13] or 1,6-hexandiamine [14,15,16,17]. Usage of ethylenediamine can even damage the polymer substrate [13].

Plasma treatment in electrical discharges is another possible way to create nitrogen functionalities on a polymer surface using nitrogen gas [18,19,20,21,22,23,24] or ammonia gas [25,26,27]. A problem with plasma treatment is the poor long-term stability of the surface functional groups because they undergo a hydrophobic recovery [28]. Coating with thin plasma polymer (PP) films via plasma-enhanced chemical vapor deposition (PECVD) is considered as the most stable modification method for introducing nitrogenated species to the surface. PECVD has already been applied in many studies to improve the biocompatibility of artificial polymers for various cell types, e.g., osteosarcoma cells [29,30,31], endothelial cells [32], fibroblasts [33,34,35], myoblasts [36,37], or adipose-derived stem cells [38]. Although PPs are characterized by a complex chemical structure, PECVD can be easily tuned by choosing suitable reactive gases or monomers and by appropriate conditions of the plasma deposition process (e.g., pressure, discharge power or the monomer flow rate). PECVD is therefore an ideal method for obtaining PPs with desirable functional properties in one simple step.

Allylamine (AA) has been the main monomer used for amine-rich plasma polymerization [30,32,34,35]. However, this compound is not the best choice for biomedical applications because of its toxicity [39]. Our previous publication [40] showed that a non-toxic AA isomer called cyclopropylamine (CPA) can be used for the deposition of amine-PP layers with a relatively high amount of amine groups (up to 10 at.%). The nitrogen content in PPs decreased with rising applied power because of higher CPA fragmentation, which also enhanced the water stability of the PPs as a consequence of higher cross-linking. Under optimal conditions, only 2% of the thickness of the amine-PP layer was lost after water immersion. On the other hand, a significant thickness loss was observed for PPs that were prepared at low applied power and therefore retained a high amount of amine groups. These findings were taken into account when choosing the plasma conditions for amine-PP deposition in the present study focused on acquiring more information about the biocompatibility and immunogenicity of amine-PP-coated polycaprolactone nanofiber (PCL NF) mats intended for the manufacture of artificial vascular grafts.

FDA-approved PCL is a promising polymer for tissue engineering because of its biodegradability, relatively low cost, easy processing, good mechanical properties, non-toxicity, and low immunogenicity [41]. In addition, the nanostructured morphology of PCL NFs, created by electrospinning, shows structural similarity with the fibrillary structure of the extracellular matrix of blood vessels. Under normal conditions, most vascular smooth muscle cells (VSMCs) in mature vessels display a differentiated contractile phenotype with a low rate of proliferation, migration, and production of extracellular matrix [42]. The complex morphology of PCL NFs should therefore better mimic in vivo conditions than the smooth surface of the polystyrene (PS) culture dishes that are widely used for in vitro experiments. The rough nanostructured morphology of PCL NFs can also decrease the proliferation rate of VSMCs [43,44,45]. This is desirable, as the excessive proliferation of VSMCs accompanied by inflammation is considered to be the main cause of intimal hyperplasia of grafts in vivo, resulting in graft restenosis [46,47].

Since coating polymers with amine-PPs is promising for the construction of artificial vascular grafts, we aimed to obtain complex information about the behavior of VSMCs on materials of this type with various chemical structures and morphologies (i.e., PS culture dishes and PCL NFs). Our previous studies [36,37] showed good biocompatibility of amine-PP coatings evaluated by mouse myoblasts 24 h after seeding. However, very few assessments of longer in vitro cultivation can be found in the literature. Moreover, no investigation of amine-PP coatings intended for the manufacture of vascular grafts and evaluating the adhesion and proliferation of vascular smooth muscle cells has yet been performed. In addition, the present study includes an assessment of the potential immunogenicity of amine-PP-coated PCL NFs.

## 2. Results

### 2.1. Material Characterization of Amine-PP-Coated PS Dishes and Amine-PP-Coated PCL Nanofibers

**Figure 1:**
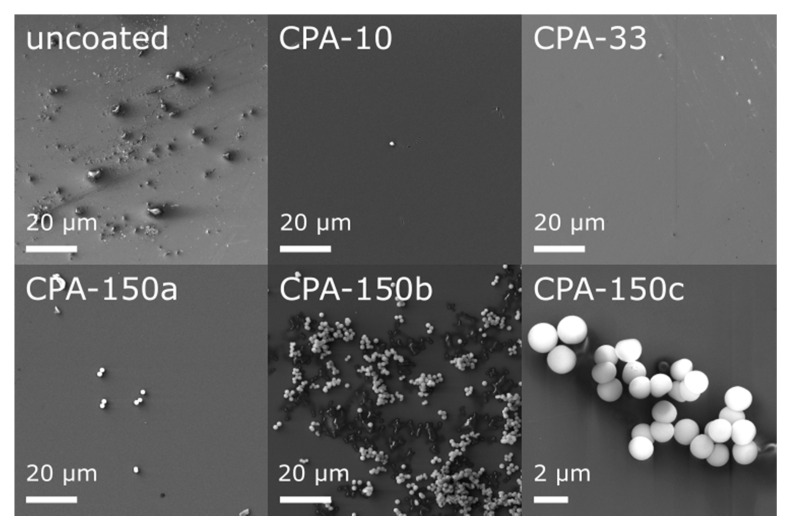
Micrographs of the surface of uncoated and plasma polymer (PP)-coated polystyrene (PS) culture dishes after 7-day exposure to the culture medium (without cells). CPA-10, CPA-33, CPA-150: PPs were deposited at the average RF power (*P*_av_) of 10, 33, and 150 W, respectively. CPA-150a, CPA-150b, CPA-150c show different regions with different magnifications on the CPA-150 sample.

Prior to an in vitro biocompatibility evaluation of the investigated materials, we characterized their morphology and their chemical structure. In our recent study [48], we have already reported that the topography of all used standard PS culture dishes (uncoated and also amine-PP-coated) evaluated by atomic force microscopy exhibited rather low roughness and was quite flat. This was apparent from the roughness rms values—5.7 ± 0.9 nm for uncoated dishes, 3.4 ± 0.8, 5.4 ± 1.2, and 4.4 ± 0.8 nm for dishes coated at the average RF power (*P*_av_) of 10, 33, and 150 W, respectively. However, high-resolution SEM showed that bumps of various sizes and amounts appeared locally on the otherwise smooth inner surface of uncoated dishes (Figure 1—uncoated). These features completely disappeared after the deposition of thin PP films, as can be seen from the micrographs of three amine-PPs deposited at *P*_av_ = 10, 30, and 150 W (Figure 1—CPA-10, CPA-33, CPA-150). Regardless of the deposition conditions, the PP surface was smooth, without cracks and pinholes even after 7-day exposure to the culture medium (without cells). An exception was the areas with spherical microparticles (Figure 1—CPA-150a–c), 1.5 ± 0.1 μm in diameter, formed on top of PP films. The number of microparticles rose with increasing *P*_av_; they rarely occurred in the case of CPA-10 or CPA-33 but they occurred in bigger numbers for CPA-150 (Figure 1—CPA-150). There was not an equal number of microparticles over the whole surface of CPA-150—there were local variations. On the same PS dish, we could find places with a small number of individual particles (Figure 1—CPA-150a) as well as areas with a big number of aggregated particles (Figure 1—CPA-150b). Almost no microparticles were observed on PP-coated PCL nanofibers.

SEM also proved that after optimizing the electrospinning process we were able to prepare homogeneous PCL NFs with a diameter of 130 ± 40 nm. The electrospun mats were composed of randomly oriented NFs with sporadically present beads (Figure S1—uncoated in Supplementary Materials). The nanofibrous mats coated with amine-PP thin films (Figure S1) showed no observable differences among the deposition conditions that were used. The morphology of the nanofibers remained preserved, and was neither influenced nor damaged by the interaction with the plasma discharge, as had already been proved in our previous study [49]. The nanofiber diameter increased from 130 nm in the case of pristine nanofibers to 210 ± 50 nm for plasma-coated nanofibers, regardless of the deposition conditions, since the deposition time was optimized to obtain the same layer thickness in all cases.

The elemental composition of the uncoated PS culture dishes, obtained by X-ray photoelectron spectroscopy (XPS), revealed the presence of oxygen, in addition to the carbon expected from the pure PS chemical formula. The amount of oxygen, 11.3%, confirmed some kind of PS surface treatment performed by the manufacturer in order to increase its attractiveness for cell adhesion and growth. Unfortunately, the manufacturer only declares that PS dishes underwent a kind of surface treatment but the type or the process of the used treatment is not specified. The results of our XPS analysis indicate the use of oxygen plasma, which is widely used for modifying polymer materials, generally resulting in the formation of hydrophilic, oxygen-containing functional groups. This assumption was supported by high-resolution C1s spectra deconvolution (Figure S2—PS dish) revealing the functional groups, which corresponded to a polymer material modified by oxygen plasma treatment [50,51,52,53].

The elemental composition of PCL expected from the chemical formula is 25% oxygen and 75% carbon, which was in good agreement with the O and C percentages determined for the as-prepared PCL NFs from XPS—25.1% oxygen and 74.9% carbon. The functional groups from the C1s environment of pristine PCL NFs (Figure S2—PCL) were specified with respect to the data from the literature [36,50].

**Figure 2:**
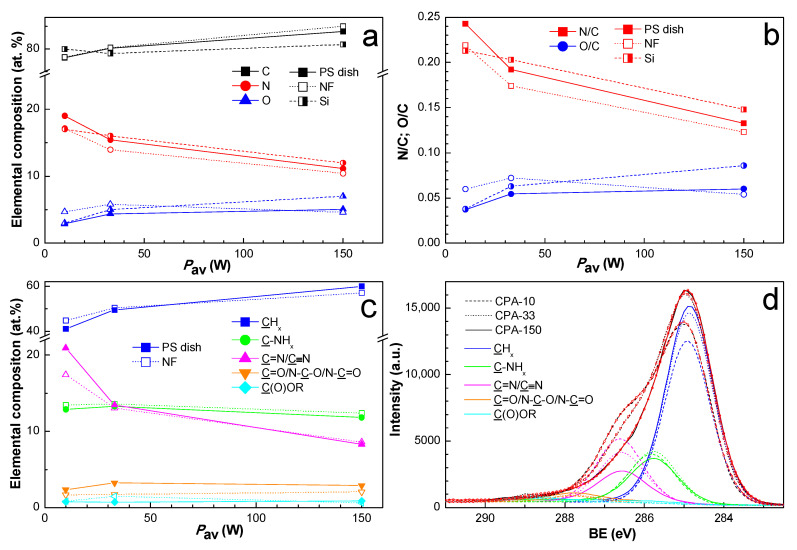
XPS chemical composition of PP-coated PS dishes and PP-coated polycaprolactone nanofibers (PCL NFs) aged two weeks supplemented with the chemical composition of PP-coated Si from our previous publication [37]: (**a**) elemental composition, (**b**) N/C and O/C ratios, (**c**) concentration of identified functional groups from C1s fitting, and (**d**) high-resolution C1s spectra with identified functional groups for PP-coated PCL NFs. PS dishes are denoted by full marks, PCL NFs are denoted by empty marks, and Si is denoted by half-full marks. CPA-10, CPA-33, CPA-150: PPs were deposited at the average RF power (*P*_av_) of 10, 33, and 150 W, respectively.

Since the two-week-old coated PS dishes and also the two-week-old coated PCL NFs were used for cell-related tests, the chemical composition of the amine-PPs was also investigated for two-week-aged samples in order to ensure data correspondence. The elemental structure and the chemical composition of PPs prepared from CPA under given plasma conditions have already been thoroughly described elsewhere [36,37,49]. The nitrogen to carbon (N/C) ratio (Figure 2b) decreased with growing *P*_av_ from 0.24 to 0.13, a trend which is known from the literature [37,40]. We observed a discrepancy in the N/C ratio among different substrates. Contrary to expectations, the oxygen concentration (Figure 2a) did not differ significantly for amine-PPs aged for two weeks in the present study in comparison with specimens characterized in the shortest possible time after preparation in our previous works [36,37,49]. This finding proved the efficiency of storage at −20 °C, which slowed down the oxidation processes to a remarkable extent.

In order to identify the functional groups contained in amine-PPs, the C1s peak was fitted with a sum of five components: aliphatic hydrocarbon groups (CH_x_) at 285.0 eV, amine groups bonded to carbon (C-NH_x_) at 285.9 eV, imine or nitrile groups (C=N/C≡N) at 286.7 eV, carbonyl or amide groups (C=O/N-C=O/N-C-O) at 287.9 eV, and ester groups (C(O)OR) at 288.9 eV. The full width at a half maximum (FWHM) for all components was set to 1.36 ± 0.04 eV, where such low FWHM values reflect the finely defined chemical structure of amine-PP. The binding energy values (BE) were determined according to the data stated in the literature [40,50,54]. The C1s spectra with identified functional groups are presented just for coated PCL NFs in Figure 2d. The atomic percentage of functional groups for the coated PS dishes and for the coated PCL NFs differed only slightly, and the values are presented in Figure 2c. The content of CH_x_ increased with rising *P*_av_ at the expense of nitrogen-containing groups, which is in good agreement with the general drop in nitrogen with growing *P*_av_, and *vice versa*. The total amount of nitrogen-containing groups decreased with rising *P*_av_ from 36.2 to 23.0 at.%. For optimal conditions (CPA-33), the amount of C-NH_x_ was approximately equal to the amount of C=N/C≡N, and the C-NH_x_ to C=N/C≡N ratio was lower than 1 in the case of low *P*_av_ (CPA-10). However, the same ratio was higher than 1 for high *P*_av_ (CPA-150). In general, the concentration of oxygen-containing groups was relatively low, and it did not vary under different conditions. The presence of a small amount of ester group may have originated from the substrates themselves (PS and PCL). However, it is more likely an outcome of post-plasma oxidation processes because of ageing in the PPs, since the ester group was also observed on substrates with no content of esters, e.g., on silicon substrates.

**Figure 3:**
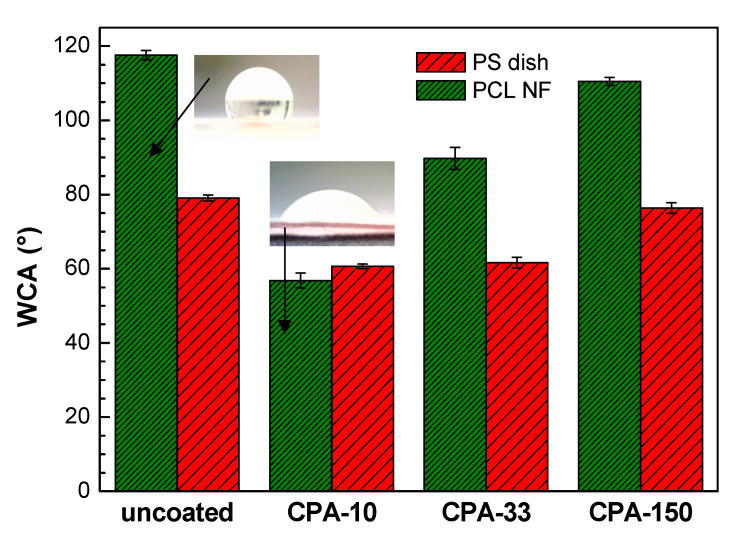
Water contact angle (WCA) values on uncoated and PP-coated PS dishes and on PCL NFs aged for two weeks. CPA-10, CPA-33, CPA-150: PPs were deposited at the average RF power (*P*_av_) of 10, 33, and 150 W, respectively.

The wettability of the PS dishes and of the PCL NFs substrates changed after the deposition of PP thin films containing functional polar groups. This is represented by water contact angle (WCA) values in Figure 3. In the case of coated PS dishes, the WCA was reduced by about 20° for samples coated at lower *P*_av_ (CPA-10 and CPA-33) in comparison with the uncoated PS dish. It should be mentioned that, despite the different amounts of nitrogen functional groups, the WCA obtained for these two conditions is almost equal. The decrease in WCA was almost negligible for the highest *P*_av_ (CPA-150). Similarly, for PCL NFs coated at the highest *P*_av_, the surface retained its hydrophobic properties with a slight decrease in WCA in comparison with pristine PCL NFs. In the case of PCL NFs coated at lower *P*_av_, the WCA was significantly decreased. A hydrophilic surface was therefore obtained, which is in good agreement with the content of nitrogen functional groups given by XPS. Images of water droplet shapes on all studied surfaces can be found in Figure S3 in Supplementary Materials.

### 2.2. In Vitro Biocompatibility of Amine-PP-Coated PS Dishes

**Figure 4:**
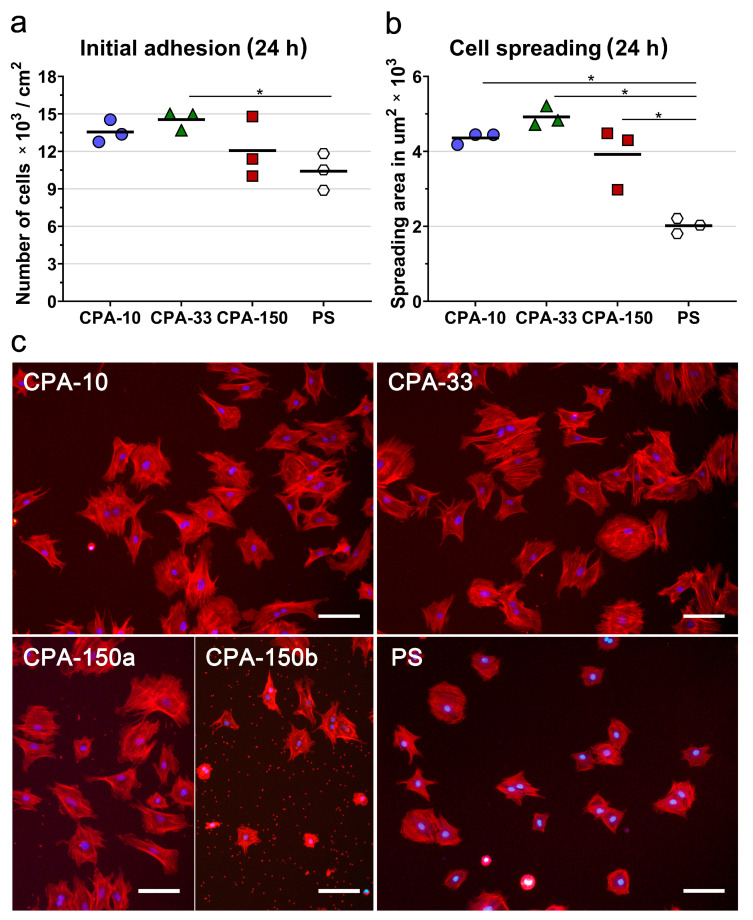
Initial adhesion of VSMCs on PS culture dishes coated with PP films deposited at the average RF power (*P*_av_) of 10, 33, and 150 W (labelled as CPA-10, CPA-33, and CPA-150, respectively): (**a**) number of adhered cells, (**b**) cell spreading areas, and (**c**) cell morphology 24 h after seeding. (**a**,**b**) The dots represent individual biological samples. Each dot depicts the arithmetic mean calculated (**a**) from cell numbers obtained from 15 pictures per each biological sample or (**b**) from spreading areas of a minimum of 120 cells per each biological sample. The bold central lines show the arithmetic mean of biological samples. Statistically significant differences (*p* ≤ 0.05) are marked by horizontal lines with asterisks connecting the differing samples. (**c**) Microphotographs were taken by an epifluorescence Olympus IX-71 microscope. Cell nuclei are visualized by Hoechst #33258 (blue), while the cell membrane and the actin cytoskeleton are visualized by Phalloidin-TRITC (red). The scale bar depicts 100 µm.

First, we evaluated the initial adhesion of VSMCs, i.e., the initial attachment of the cells to the surfaces of the investigated PP-coated PS dishes 24 h after seeding, which was observed to be a typical initial adhesion time for VSMCs. The initial adhesion of VSMCs (assessed by the number and by the spreading area of the cells 24 h after seeding) showed that all PP-coated PS dishes induced faster initial adhesion of VSMCs than the uncoated PS dish. Samples deposited at 33 W (CPA-33) revealed significantly higher cell number values than the uncoated PS dishes (Figure 4a). Although it was not proven to be statistically significant, samples deposited at the average power of 10 W (CPA-10) and 150 W (CPA-150) also exhibited higher cell numbers than the uncoated PS dishes (Figure 4a). The initial spreading of VSMCs (i.e., the area of cells attached to the surface of the material 24 h after seeding) was two times larger on all three PP-coated PS dishes than on the reference uncoated PS dishes (Figure 4b,c).

The morphology of VSMCs cultured on all amine-PP-coated dishes for 24 h was mostly polygonal with already well-spread cells with numerous protrusions and already well-developed actin stress fibers (Figure 4c). This favorable pattern was markedly more apparent on all amine-PP-coated dishes than on the uncoated PS dishes, where a larger number of still rounded less-spread cells was observed (Figure 4b,c). Interestingly, a microscopic evaluation of sample CPA-150 revealed heterogeneous coverage by VSMCs, with the cell morphology varying from well-spread to less-spread (Figure S4a). This phenomenon was attributed to the presence of a larger amount of microparticles unevenly distributed on the surface of the sample CPA-150. While the parts of the sample with a smaller amount of microparticles were comparable with CPA10 and CPA-33 (Figure 4c—CPA-150a), the parts with a higher occurrence of particles showed lower cell densities and also a lower cell spreading with a larger number of rounded cells (Figure 4c—CPA-150b). However, the overall performance of the cells (cell numbers and spreading) did not differ significantly from the cells cultured on CPA-10 and on CPA-33 (Figure 4a,b).

**Figure 5:**
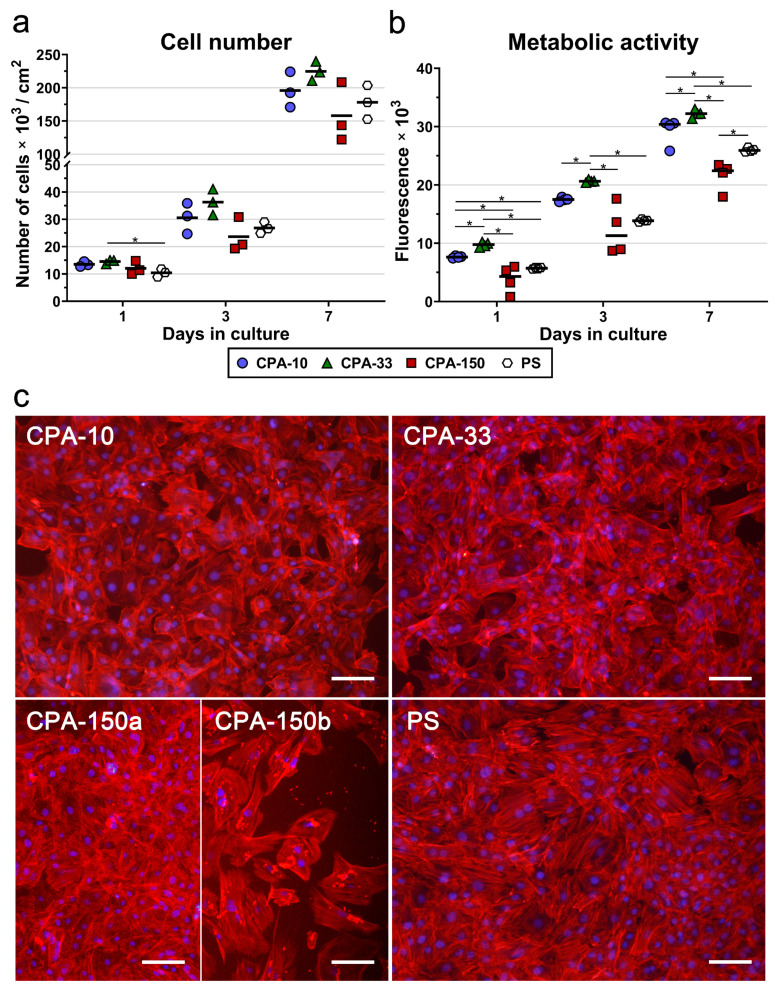
(**a**) Proliferation, (**b**) metabolic activity, and (**c**) morphology of VSMCs cultured on PS culture dishes coated with PP films, deposited at the average RF power (*P*_av_) of 10, 33, and 150 W (labelled as CPA-10, CPA-33, and CPA-150, respectively), evaluated within 7 days of cultivation. (**a**,**b**) The dots represent individual biological samples. Each dot depicts the arithmetic mean calculated (**a**) from cell numbers obtained from a minimum of 15 pictures per biological sample or (**b**) from three pipetting replicates per biological sample. The bold central lines show (**a**) the arithmetic mean and (**b**) the median of biological samples. Statistically significant differences (*p* ≤ 0.05) are marked by horizontal lines with asterisks connecting the differing samples. (**c**) Images of the cells were captured 7 days after seeding by an epifluorescence Olympus IX-71 microscope. Cell nuclei are visualized by Hoechst #33258 (blue), while the cell membrane and the actin cytoskeleton are visualized by Phalloidin-TRITC (red). The scale bar depicts 100 µm.

Similarly to the initial adhesion, VSMCs cultured for 3 and 7 days on samples CPA-10 and CPA-33 reached higher cell numbers than the cells grown on sample CPA-150 and on the reference PS. However, the differences among the samples were not proven to be significant. Sample CPA-150 again exhibited the lowest cell densities among all PP films, together with the greatest variance within the replicates (Figure 5a), caused by the heterogeneity of the cell performance between areas with a lower occurrence of particles and areas with a greater occurrence of particles (Figure S4b and Figure 5c—CPA-150a and b). A microscopic evaluation proved that cells cultured on all samples, including the reference PS, reached semi-confluence after 7 days of cultivation (Figure 5c).

In order to prove significant differences among the amine-PP-coated PS dishes, we also decided to evaluate the metabolic activity of VSMCs during the 7-day long cultivation. As expected, the overall trend of the metabolic activity was well-correlated with the results for cell numbers. For all investigated time intervals, the cells cultured on the CPA-33-coated dish displayed significantly higher metabolic activity than the cells cultured on the other PP-modified PS dishes, and also on the control unmodified PS (Figure 5b). The values for sample CPA-10 were higher than the values for CPA-150 and for the reference PS dish. However, statistically significant differences were proven only for day 1 and for day 7 (in comparison with CPA-150) and for day 1 (in comparison also with the PS dish). As has been described above, sample CPA-150 again exhibited the lowest metabolic activity values with the highest variance within the replicates.

### 2.3. In Vitro Biocompatibility and Non-Immunogenicity of Amine-PP-Coated PCL Nanofibers

**Figure 6:**
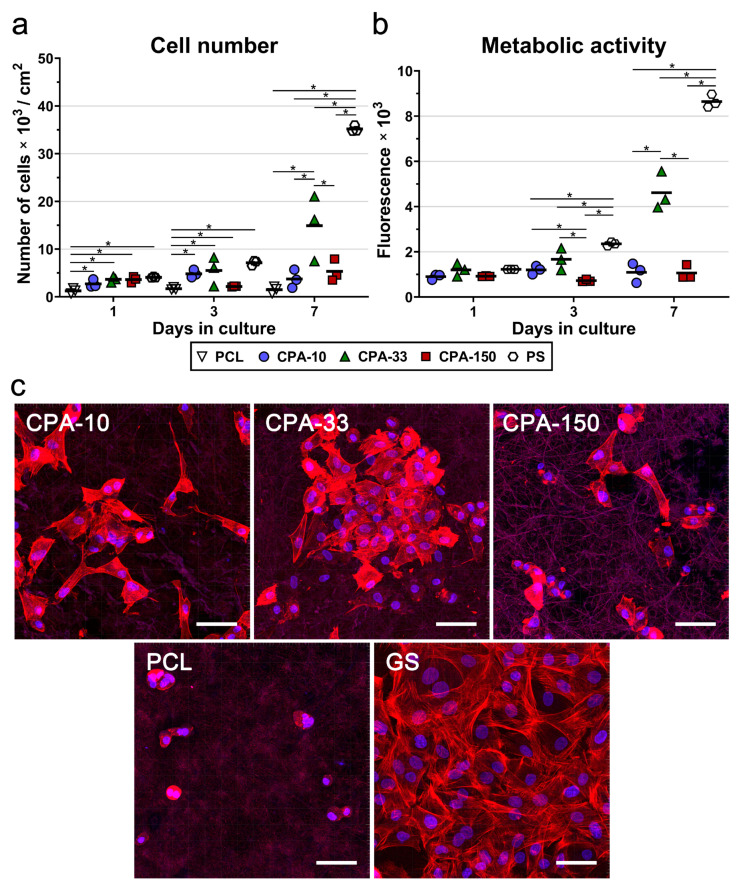
(**a**) Proliferation, (**b**) metabolic activity, and (**c**) morphology of VSMCs cultured on amine-PP-coated PCL NFs, deposited at the average RF power (*P*_av_) of 10, 33, and 150W (labelled as CPA-10, CPA-33 and CPA-150, respectively), evaluated within 7 days of cultivation. (**a**,**b**) The dots represent individual biological samples. Each dot depicts the arithmetic mean calculated (**a**) from cell numbers obtained from a minimum of 10 pictures per biological sample or (**b**) from three pipetting replicates per biological sample. The bold central lines show the arithmetic mean of biological samples. Statistically significant differences (*p* ≤ 0.05) are marked by horizontal lines with asterisks connecting the differing samples. (**b**) Pristine PCL NFs are excluded from the graph because of their non-specific reaction with the resazurin assay. (**c**) Images of the cells were captured 7 days after seeding by a confocal Leica TCS SP8 microscope. Cell nuclei are visualized by Hoechst #33258 (blue), while the cell membrane and the actin cytoskeleton are visualized by Phalloidin-TRITC (red). GS—glass coverslips. The scale bar depicts 50 µm.

To confirm the best performance of the amine-PP film deposited at the average RF power of 33 W (CPA-33), the same PP films (CPA-10, CPA-33, and CPA-150) were deposited on PCL nanofiber mats. The cell numbers, including the numbers of initially adhered cells, and the metabolic activity of the VSMCs were evaluated during 7-day long cultivation. The results of the initial adhesion of VSMCs on pristine and amine-PP-coated PCL NFs proved that all investigated modifications improved the adhesion of the cells to the nanofibers. The number of initially attached cells on the PP-coated PCL NFs was about 2–3 times higher than the number of cells found on the pristine PCL NFs 24 h after seeding (Figure 6a). The values for all coated PCL NFs were comparable to the cell numbers found on the reference PS. As in the case of the data obtained from the PP-coated dishes, the VSMCs cultured for 3 days on samples CPA-10 and CPA-33 reached higher cell numbers than the cells grown on sample CPA-150 and on the pristine PCL NFs (Figure 6a). However, the only significant difference among the samples was found between the pristine PCL NFs and the PP-coated PCL NFs, but not among the PP-coated PCL NFs. Significant differences among the PP-coated samples were found after 7 days of cultivation. The cells grown on CPA-33 reached about three times higher cell numbers than the cells cultured on CPA-10 and on CPA-150 (Figure 6a).

The assessment of VSMC metabolic activity (Figure 6b) confirmed the clear preference of these cells for CPA-33 over CPA-150 (on days 3 and 7), and over CPA-10 (on day 7). Pristine PCL NFs were excluded from the metabolic activity analysis because of their non-specific reaction with resazurin, which caused a false positivity. The amine-PP-coated PCL NFs did not show any non-specific reaction (i.e., any non-specific conversion of resazurin into resorufin by cell-free samples), as was confirmed by a good correlation with the cell number values (Figure 6b). Figure 6a,b show that the cells cultured on pristine PCL NFs and also on NFs coated with CPA-10 and CPA-150 films did not proliferate over a period of 7 days. The cell number values, and also the values for the metabolic activity on these samples, remained the same from day 1 to day 7 after seeding. These values increased with time only on the nanofibers coated with CPA-33 films.

A microscopic evaluation showed that the cells cultured on pristine PCL NFs were poorly spread and had a rounded morphology without any developed cytoskeleton (Figure 6c). All amine-PP coatings improved the cell spreading and the cytoskeleton development in VSMCs. The cells cultured on CPA-10 and on CPA-33-coated PCL had a better-spread morphology than the cells grown on CPA-150-PCL (Figure 6c). It is apparent that the cell spreading is inversely correlated with the WCA values (Figure 3). Thus the poor spreading of VSMCs cultured on pristine PCL NFs and on CPA-150-modified PCL NFs can be mainly attributed to the low wettability of these samples.

**Figure 7:**
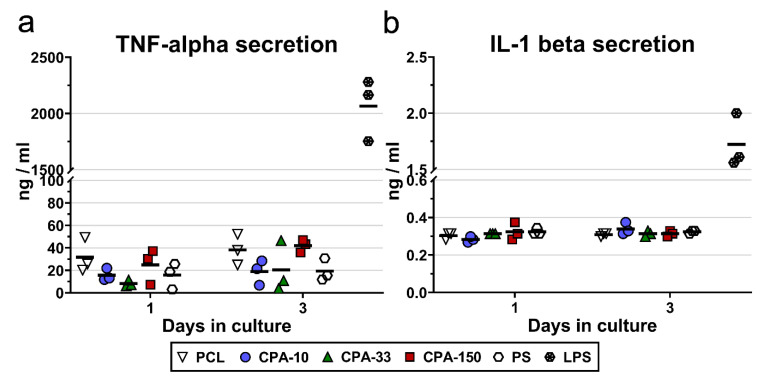
Secretion of (**a**) TNF-alpha and (**b**) IL-1-beta into the culture media by RAW 264.7 cells cultured on amine-PP-coated PCL NFs, deposited at the average RF power (*P*_av_) of 10, 33, and 150 W (labelled as CPA-10, CPA-33, and CPA-150, respectively), evaluated within 3 days of cultivation. The dots represent individual biological samples. Each dot depicts the arithmetic mean calculated from three pipetting replicates per biological sample. The bold central lines show the arithmetic mean of biological samples. LPS—a positive control of TNF-alpha and IL-1-beta secretion induced by incubation of RAW 264.7 cells with lipopolysaccharide (10 μg/mL for 24 h).

The potential immune activation of RAW 264.7 cells was evaluated by the amount of TNF-alpha molecules and IL-1-beta molecules secreted into the culture medium by cells cultured on PP-coated PCL NFs for 3 days. The results showed slightly increased secretion of TNF-alpha by the RAW 264.7 cells cultured on the pristine PCL NFs and on the CPA-150-coated PCL NFs (Figure 7a). However, this increase was not proven to be significant. Similarly, the amount of IL-1 beta secreted into the culture media did not differ among the evaluated samples (Figure 7b).

## 3. Discussion

Although many publications have reported a positive effect of various amine-PP coatings or treatments on the adhesion and proliferation of various cell types, the optimal concentration of the amine groups is still unclear. Some studies have claimed that the number of attached cells increased with the increasing amount of amine groups on the surface [55,56,57]. However, other studies have failed to show any correlation between the concentration of an amine group and the number of attached cells [30,35,36,37,58]. Similarly conflicting results have also been found for the effect of the amine group concentration on the proliferation of various cell types [35,57,58,59,60]. Not only the optimal concentration but also the type of functional groups is still in question. While most publications agree about the positive effect of nitrogen functional groups, some works have shown similar enhancement of bioactivity for surfaces with both oxygen and nitrogen functional groups. No specific relation between the concentration of O and N functional groups and the cell proliferation has been reported [20,22,24]. Several studies have even revealed better cell adhesion or better cell proliferation on surfaces containing oxygen functionalities than on surfaces with nitrogen functional groups [18,21,23,29]. The discrepancies among the findings presented by different research groups strongly suggest a complexity of the problem, in which the overall chemical structure comprising nitrogen and oxygen-containing groups can be more important than the exact concentration of certain groups. Moreover, other factors such as different preferences among various cell types, surface wettability, and morphology, also play an important role.

The present study has shown faster initial adhesion of VSMCs on amine-PP-coated PS dishes and on amine-PP-coated PCL nanofibers, regardless of the amine-PP chemical structure and regardless of the material surface morphology (Figure 4 and Figure 6a—day 1). Although VSMCs typically spread very slowly on standard PS culture dishes, the cells cultured on PP-coated PS dishes were already well-spread with numerous protrusions and well-developed actin stress fibers 24 h after seeding (Figure 4a). Other studies have also reported improved adhesion of various cell types to similar amine-PP coatings deposited on various flat substrates [30,31,37,48,55,61]. In the works of Finke’s group [30,31] and Mangindaan et al. [55], amine-PPs prepared from AA or ethylenediamine also induced faster initial adhesion of human osteosarcoma cells and fibroblasts, proven by higher cells numbers and larger spreading areas. Moreover, our previous publications [37,48] revealed markedly higher resistance of various cell types to trypsin treatment, suggesting stronger cell adhesion to positively charged PP coatings. A different amine polymer deposition method, namely UV grafting of CPA through a ring-opening reaction, also confirmed two times higher adhesion of three different adenocarcinoma cell lines to a CPA-modified silicon (100) hydride substrate than to an uncoated silicon control 24 h after cell seeding [61]. Not only the flat PS substrate coated by PPs but also the nanostructured PCL NFs with PPs increased the initial attachment of cells 2–3 times, reaching cell number values comparable to those of the reference PS (Figure 6a). Similar results were also found in two other publications investigating the adhesion of various cells to amine-PP-coated PCL nanofibers [36,58]. Our previous study by Manakhov et al. [36] showed a 2-times higher amount of total ATP, which is an indirect measurement of cell numbers, and also 2-times higher spreading areas of mouse myoblasts seeded on amine-PP-coated PCL NFs than of the cells cultured on uncoated NFs 24 h after seeding. A second study confirmed improved adhesion of human fibroblasts to CPA-modified PCL NFs by increased metabolic activity and also by larger cell spreading areas assessed 24 h after seeding [58].

The improved initial adhesion of cells to amine-PP coatings observed in the present study and also in the publications mentioned above can be attributed to a relatively high presence of amine groups, which are often associated with the enhancement of materials bioactivity and biocompatibility [9,15,26]. Indeed, the positively charged amine groups on the material surface create electrostatic interactions with the negatively charged cell-adhesive proteins present in the serum supplement of standard cell culture media, such as fibronectin, vitronectin, collagen, laminin, or fibrin, which facilitate cell adhesion to this surface [5]. Moreover, the positively charged amine groups can also directly form electrostatic interactions with the negatively charged cell surface. This charge-mediated adhesion is a non-specific, i.e., non-receptor-mediated, type of cell adhesion. Studies by Geiger’s group [62,63] described the importance of negatively charged hyaluronan, which in the early stage of cell adhesion forms a thick pericellular coat surrounding most cell types. This is usually followed by specific receptor-mediated cell adhesion. Another publication discusses the importance of negatively charged glycocalyx, which is another type of pericellular coat composed of glycoprotein and glycolipid, in non-specific charge-mediated adhesion to amine groups [64]. Since this non-specific adhesion did not require an interaction between a particular adhesion receptor on the cell surface (i.e., specific integrin) and its extracellular binding partner (e.g., fibronectin, vitronectin etc.,), it can occur much faster, which we also observed in the present study. Indeed, a recent study by Hasan et al. [65] has also reported faster initial adhesion of mouse fibroblasts to amine-rich surfaces than to carboxyl-rich or octyl-rich surfaces. However, it should be pointed out in this context that non-specific charge-mediated cell adhesion through amine groups is not capable of delivering a specific signal to cells in order to stimulate their subsequent proliferation. If this non-specific adhesion is not accompanied or followed by a sufficient level of specific cell adhesion, usually mediated by integrin adhesion receptors on the cells, the anchorage-dependent cells cannot grow, and they even undergo apoptosis [48,66].

Only a limited number of publications have evaluated the effect of amine-PP coatings on cell proliferation. As would be expected from the positive results for initial adhesion, amine-PP coatings deposited on various substrates from AA, CPA or diethylene glycol dimethyl ether precursors also improved the growth of human fibroblasts during the 7-day long cultivation [33,35,58,60]. Similarly, the proliferation rate of adipose-derived stem cells cultured on pyrrole-modified PS dishes increased in correlation with the increasing concentration of nitrogen and nitrogen-related functional groups [38]. However, other publications have revealed quite opposite findings that show reduced proliferation of various cell types cultured on amine-PP coatings prepared from AA, a methane/nitrogen mixture or from CPA [29,32,48]. Similarly ambiguous results were observed in the present study. Although all CPA coatings improved the initial adhesion of VSMCs, the longer period of cultivation of VSMCs on CPA-modified samples showed clearly that these cells proliferate preferentially on a CPA-33 coating, regardless of the chosen deposition substrate (PS or PCL NFs). This coating exhibits 1.5 at.% of primary amines [67], which is about one half of the concentration of the coating with the highest amount of primary amine groups—2.9 at.% of NH_2_ for CPA-10 [36]. However, the different proliferation activity of VSMCs on all three tested CPA coatings cannot be attributed simply to the different concentration of amine groups on these surfaces, because the absolute amount of these groups (primary or secondary) was similar for all PPs in the present study and was equal to ~13 at.%. Other plentifully present groups were imines/nitriles, the concentration of which in the CPA films decreased with rising *P*_av_ from 20 to 8 at.%. Coated substrates also contained below 3 at.% of amide, carbonyl or ester groups. Nevertheless, there is no direct connection between the number of primary amine groups, or possibly other functional groups, and the clear preference of VSMCs for CPA-33-coated substrates. This shows that also other criteria, and not only the chemical composition of PPs, affect the biocompatibility of amine-PP coatings.

Another critical factor affecting the cell behavior is the stability of the coating in a water environment. Our previous study [37] showed that increasing discharge power of CPA plasma polymerization from 5 W to 150 W resulted in a decreased amount of amine groups, and simultaneously increased the water stability from 23% to only 2.7% of the thickness loss. The number of mouse myoblasts evaluated 24 h after seeding revealed that the cells preferred coatings with greater water stability over coatings with a higher content of amine groups [37]. Moreover, the present study has shown that VSMCs preferred CPA-33-coated NFs, despite their relatively high WCA (90°), over CPA-10-coated NFs with WCA values below 60° (Figure 3), which are considered as optimal for cell culture [68,69]. The higher proliferation and metabolic activity of VSMCs cultured on CPA-33 coatings than on CPA-10, regardless of the substrate, therefore has to be explained by the better stability of CPA-33 coatings in a water environment. Although the CPA-150 coating has been shown to have the highest water stability [37], its wettability decreased only slightly from 79° to 76° on PS and from 118° to 111° on PCL NFs (Figure 3). High WCAs can be associated with the lowest amount of polar amine groups and other hydrophilic groups. While the value of 111° fully explains the poor spreading and ceased proliferation of VSMCs cultured on CPA-150-coated PCL NFs, the contact angle of 76° is probably only a partial reason for the lower proliferation of cells cultured on CPA-150-coated PS. A more important factor can be a different surface morphology of CPA-150-coated PS, i.e., a big amount of microparticles (Figure 1—CPA-150).

It was shown by Santos et al. [70] that well-shaped substrates (e.g., PS dishes) can behave as particle collectors. In dusty plasmas, negatively charged nanoparticles are electrostatically kept in the positive plasma potential and they levitate in vertical equilibrium positions near the plasma sheath above the flat substrate. Modification of the substrate geometry from flat to well-shaped leads to the entrapment of an increased number of particles in well-like substrates. This particle collection is likely driven by two main factors. First, the positive plasma potential can expand inside the wells, facilitating the confinement of particles. Second, the formation of a plasma sheath around the wells results in an electric field profile that acts as an electrostatic converging lens, concentrating the particles around the central well axis. The input power and working pressure influence the particle number and their aggregation. Like Santos et al. [70], we can argue for the observation of a big amount of heterogeneously distributed microparticles on the CPA-150 coating on well-shaped PS dishes and not on flat PCL NFs.

VSMCs adhered preferentially on the parts of CPA-150-coated dishes with a smaller amount of microparticles, and a large amount of microparticles negatively affected their proliferation. Similar results were observed by Chen et al. [71], when comparing the cytotoxicity of MoS_2_ microparticles with the cytotoxicity of MoS_2_ films. The solid MoS_2_ films showed no signs of cytotoxicity for six different cell types, but the presence of microparticles caused a concentration-dependent decrease in proliferation of all cell types that were used. A considerable number of other publications have reported concentration-dependent cytotoxic effects of silicon, SiO_2_, and ZnO microparticles [72,73,74], while SiO_2_ and ZnO in the form of films demonstrated good biocompatibility without having a negative effect on cell proliferation [75,76,77,78]. Moreover, an in vivo study performed on rodents (mice and rats) by Taylor et al. [79] revealed chronic inflammation and gross fibrin formation after the subcutaneous injection of kafirin microparticles 1–5 µm in diameter, whereas no abnormal inflammatory reaction was found after the subcutaneous implantation of films prepared from the same kafirin microparticles. According to two other studies [73,74], not only the concentration but also the size of the microparticles strongly affects their cytotoxicity. Porous silicon microparticles (PSi) with a diameter of 1–25 µm and thermally oxidized porous silicon microparticles (TOPSi; 1–25 µm in diameter) displayed markedly higher cytotoxicity than either larger microparticles (25–75 µm for PSi and 17 µm for TOPSi) or much smaller TOPSi nanoparticles (110 nm in diameter). Our CPA-150 particles have an average diameter of 1.5 µm, i.e., similar to the dimensions of the cytotoxic microparticles discussed above. The highest presence of microparticles observed on CPA-150-coated PS dishes is therefore probably more responsible for the slowest proliferation of VSMCs than the lowest amount of amine groups together with the lowest surface wettability among the amine-PP-coated dishes.

Besides the presence of microparticles, the morphology of culture substrates is also known to be an important parameter affecting cell adhesion and proliferation [5,80]. The trend of the proliferation and metabolic activity of VSMCs was similar for amine-PP-coated PS dishes and for amine-PP-coated PCL NFs, with the highest cell densities found on amine-PPs prepared at *P*_av_ = 33 W. The values for CPA-33-coated dishes were slightly higher than for uncoated PS dishes (Figure 5). However, the CPA-33-coated NFs successfully lowered the proliferation rate of VSMCs about two times in comparison with the CPA-33-coated dishes and the reference uncoated PS wells, while they still supported continuous proliferation of VSMCs during the 7-day-long cultivation. (Figure 6). As has already been explained in the introduction to our study, a high proliferation rate of VSMCs cultured on materials intended for vascular grafts should be avoided because it is responsible for intimal hyperplasia of grafts in vivo, resulting in graft restenosis [46]. Importantly, the evaluation of TNF-alpha and IL-6 beta secretion by RAW 264.7 macrophages (Figure 7) revealed no signs of immunogenicity of amine-PP-coated PCL NFs. This is important to know, as an inflammatory reaction can also inhibit cell proliferation and can result in intimal hyperplasia of grafts in vivo [46,47]. In accordance with our results, many studies have also shown slower proliferation of VSMCs cultured on various nanofibrous membranes in comparison to the reference PS dish [81,82,83,84,85]. Similarly, studies comparing nano-patterned or micro-patterned polymers with the same polymers with a flat morphology revealed slower proliferation of VSMCs cultured on substrates with a rougher structure [43,44,45]. It is generally known that material surface roughness can inhibit cell proliferation and can support the differentiation and phenotypic maturation of cells [5]. This is true mainly for micron-scale and submicron-scale roughness. Although polymeric fibers prepared by electrospinning, including our PCL NFs, are generally referred to as nanofibers, they are in fact submicron-scale fibers, because their diameter is usually more than 100 nm. The restricted proliferation activity of VSMCs on our newly developed amine-PP-coated PCL NFs makes this novel biomaterial promising for the construction of small-diameter vascular grafts resistant to restenosis, which are still lacking in the market. At the same time, the VSMCs on our biomaterial are metabolically active and well-spread. They are therefore viable, as required for the proper functioning of vascular grafts mimicking physiological blood vessels in vivo, in which VSMCs are an important cell component ensuring contractility of the vessel. In advanced blood vessel replacements, which are still under development, the VSMCs are utilized for reconstruction of the *tunica media*, while the vascular replacements currently used in clinical practice are usually cell-free, or are endothelialized at the most [4]. Our earlier study revealed that our newly developed amine-PPs provided good support for the adhesion and subsequent growth of vascular endothelial cells, which adhered predominantly by the specific receptor-mediated mechanism [48]. Mature endothelial cells in the form of a confluent semi-permeable layer are known to keep VSMCs in a quiescent, non-proliferative, differentiated contractile phenotype, as required for blood vessel replacements [5].

Two other factors besides the substrate morphology must be carefully taken into account when comparing amine-PP-coated dishes with amine-PP-coated NFs. First, the chemical composition of amine-PPs can be different on planar substrates, such as standard cell culture dishes, than on non-planar substrates, such as NFs. Manakhov et al. [49] reported a difference in the elemental composition of CPA-33 prepared on planar Si substrates and on PCL NFs, which was attributed to a substantially different substrate morphology. Similarly, the N/C ratio for CPA-10 and CPA-150 PPs on Si substrate [37] differed from the results obtained on PCL NFs [36]. This ratio was about 0.04–0.05 lower in the case of NF substrates. The N/C ratio of amine-PPs deposited in the present study was lower by ~0.02 on nanofibrous substrates than on PS dishes (Figure 2b). Second, PS culture dishes and PCL NFs differ not only in their surface morphology but also in their stiffness. A number of studies comparing various materials with different stiffness have reported that VSMCs cultured on substrates with lower stiffness exhibited a relatively mature phenotype with a lower proliferation rate [84,86,87,88,89]. The markedly slower proliferation of VSMCs cultured on CPA-33-coated NFs than on CPA-33-coated PS dishes is therefore caused by a combination of the rougher surface morphology and the lower stiffness of PCL NFs. A weak influence can also be attributed to the lower nitrogen content.

It can be summarized that, unlike the CPA-10 and CPA-150 coating, the CPA-33 coating deposited on PS dishes showed excellent bioactivity by promoting the fastest initial cell adhesion with no negative effect on the cell proliferation, metabolic activity, or morphology. The poorer performance of VSMCs cultured on CPA-10-coated PS dishes was most probably caused by the lowest water stability of the CPA-10 coating, while the poorest performance of CPA-150-coated PS dishes can be attributed to the large amount of microparticles. Nanofibrous mats coated with CPA-33 PP provided excellent support for VSMC cultivation, with a desirable moderate proliferation rate, while ensuring continuous proliferation of VSMCs during 7-day-long cultivation. The ceased proliferation of VSMCs grown on CPA-10-coated PCL NFs was attributed to a combination of the low water stability of the CPA-10 coating and the inhibitive effect of the nanofibrous morphology of the PCL. The ceased proliferation and the poor cell spreading of VSMCs cultured on CPA-150-coated NFs was, on the other hand, attributed mainly to the low wettability combined with the smallest amount of bioactive groups (e.g., amine and other nitrogen-containing and oxygen-containing groups). It was further enhanced by the inhibitive character of the PCL nanofibrous morphology. The amine-PP coating deposited on PCL NFs at the average deposition power of 33 W (CPA-33) therefore exhibited the best combination of functional properties for promoting better initial adhesion, better cell spreading, and slower but continual proliferation of vascular smooth muscle cells.

## 4. Materials and Methods

### 4.1. Electrospinning of PCL Nanofibers

Nanofibrous membranes were prepared by electrospinning of polycaprolactone (PCL) solution. PCL pellets (M_n_ 80,000, Sigma-Aldrich, Merck, Darmstadt, Germany) were dissolved in a mixture of acetic acid (99%, Sigma-Aldrich, Merck, Darmstadt, Germany) and formic acid (98%, Sigma-Aldrich, Merck, Darmstadt, Germany) in a weight ratio of 2:1 to acquire a PCL solution of 9 wt.% concentration. After mixing, the solution was stirred at room temperature for 24 h. The electrospinning process was based on Nanospider™ technology; nanofibrous mats were manufactured using the Nanospider NS 1WS500U laboratory device (Elmarco s.r.o., Liberec, Czech Republic). Wire spinning was used for this process. The wire electrode was made from steel with a diameter of 0.2 mm. The rotation of the wire electrode was 50 mm/min. The distance between electrodes was 200 mm, and the applied potential difference between the driving electrode and the collecting electrode was 75 kV. The nanofibers were collected on the polypropylene nonwoven spunbond, which moved around the collecting electrode at a constant velocity of 60 mm/min. These experiments were carried out at laboratory temperature (25 °C) and at relative air humidity of 20%.

### 4.2. Deposition of Amine Plasma Polymer Films

The amine-PP films were deposited on standard tissue culture polystyrene dishes (TPP, Trasadingen, Switzerland, area 9.2 cm^2^) and on nanofibrous PCL membranes. The deposition procedure was carried out in a stainless steel parallel plate reactor, the scheme of which has been described in detail elsewhere [49,60]. The bottom electrode, 420 mm in diameter, was capacitively coupled to a radiofrequency (RF) generator working at a frequency of 13.56 MHz. The gases—cyclopropylamine (CPA, 98%, Sigma-Aldrich, Merck, Darmstadt, Germany) and argon (99.998%, Messer, Brno, Czech Republic)—were fed into the chamber through a grounded upper showerhead electrode 380 mm in diameter. The distance between the electrodes was 55 mm. The reactor was pumped down to 10^−1^ Pa by a rotary vane pump. The leak rate, including wall desorption, was below 0.4 sccm for all depositions.

The substrates were placed on the bottom RF electrode and were sputter-cleaned in pulsed Ar plasma for 5 min. The pulse setting was 33% duty cycle (D.C.) and 500 Hz pulse repetition frequency at 100 W applied power (*P*).

PP thin films were prepared from a CPA/Ar deposition mixture in a pulsed wave or in a continuous wave. The average RF power (*P*_av_) calculated as *P* multiplied by the D.C. was chosen as the main parameter describing the deposition conditions. The pulsed mode setting was the same as in the case of Ar pre-treatment. PP thin films were deposited at *P*_av_ = 10 W for PP with a high amount of amine groups but with poor water stability (CPA-10), at *P*_av_ = 150 W with a small amount of amine groups but with good water stability (CPA-150), and at *P*_av_ = 33 W (CPA-33), which is regarded as a good compromise between the two previous options (conditions summarized in Table 1). The Ar flow rate was set to 28 sccm and was regulated by an electronic Hastings flow controller, while the CPA vapor flow rate was set to 2 sccm by a needle valve. The pressure was kept constant at 50 Pa throughout the deposition. The deposition time was adjusted to obtain a film thickness of about 240 nm (on a silicon substrate). The coated specimens were not used for cell-related tests immediately after deposition, but were used after a time delay of two weeks. Meanwhile, they were stored in a freezer at −20 °C, which significantly slowed down their ageing [49,90].

### 4.3. Surface Characterization

The surface morphologies of uncoated and amine-PP-coated polystyrene (PS) culture dishes and PCL nanofibers (NFs) were studied by scanning electron microscopy (SEM) using a SEM LYRA3 (Tescan) microscope in secondary emission mode (10 kV acceleration voltage, 9 mm working distance). 1024 × 1024 pixel micrographs were acquired. Prior to imaging, the samples were coated with a 10-nm thick gold film deposited by RF magnetron sputtering (Leica ACE 600) in order to eliminate the charging of the surface of the sample.

X-ray photoelectron spectroscopy (XPS) for the surface (6–9 nm) chemical characterization of PP thin films on coated substrates was carried out using an Axis Supra (Kratos Analytical) spectrometer. The spectra were subsequently normalized by shifting the hydrocarbon component CH_x_ to 285.0 eV. The element atomic percentage was quantified from the high-resolution spectra of each element, and high-resolution C1s spectra were fitted in order to obtain individual components using CasaXPS software (version 2.3.19), after subtracting the Shirley-type background employing Gaussian–Lorentzian (G-L) peaks with a fixed G-L percentage of 30%. The values of the binding energies of the C environment were taken from the literature.

The water contact angle (WCA) values were measured on coated substrates using the SEE System 7.0 sessile drop technique. This enabled the solid-liquid meniscus to be observed with the use of a CCD camera. The drop snapshots were captured with a ~0.2 s delay after the droplet touched the surface, in order to ensure a correct comparison, because the shape of the water droplet changed rapidly after the contact. The WCA values were calculated based on three-point interpolation of the drop height and width from the images.

### 4.4. Cells and Culture Conditions

Vascular smooth muscle cells (VSMCs; isolated from the *tunica media* of rat aorta by an explantation method described in our earlier study ([91]; passage number 4–7) were used for evaluating the adhesion, proliferation (cell numbers), and metabolic activity of these cells. The VSMCs were cultured in Dulbecco’s modified Eagle’s medium (Gibco, Thermo Fisher Scientific, Waltham, MA, USA) supplemented with 10% fetal bovine serum (FBS; Gibco, Thermo Fisher Scientific, Waltham, MA, USA) and gentamicin (40 μg/mL; Sandoz, Novartis, Switzerland) at 37 °C in a humidified air atmosphere containing 5% CO_2_ for 1, 3, or 7 days.

Mouse macrophages of the RAW 264.7 line (Cat. No. 91062702; ECACC, Salisbury, UK) were used for assessing the potential immunogenicity of the investigated samples. The RAW 264.7 cells were cultured in RPMI 1640 medium (Gibco, Thermo Fisher Scientific, Waltham, MA, USA) supplemented with 10% FBS and gentamicin (40 μg/mL) at 37 °C in a humidified air atmosphere containing 5% CO_2_ for 1 and 3 days.

Two types of samples were used for in vitro biological evaluation: tissue culture polystyrene dishes (9.2 cm^2^, TPP, Trasadingen, Switzerland), and nanofibrous PCL membranes coated with various amine-PP films (labelled as CPA-10, CPA-33, and CPA-150 according to the average deposition power; Table 1). Amine-PP-coated and uncoated PCL membranes, cut into square samples (1.3 × 1.3 cm in size), were fixed in CellCrown inserts (Scaffdex, Tampere, Finland ) and were inserted into polystyrene 24-well cell culture plates (TPP). Uncoated PCL membranes and standard cell culture polystyrene wells were used as reference control samples for experiments with amine-PP-coated membranes, while uncoated polystyrene (PS) dishes (9.2 cm^2^; TPP) were used as a reference control for experiments with amine-PP-coated dishes.

### 4.5. An Evaluation of Cell Adhesion, Morphology, Numbers, Metabolic Activity, and Potential Immune Activation

For an evaluation of the cell numbers and the cell morphology, amine-PP-coated PS dishes and uncoated PS dishes were seeded with VSMCs in a density of 17,000 cells/cm^2^, and PP-coated as well as pristine PCL membranes were seeded with VSMCs in a density of or 8500 cells/cm^2^. PS dishes for spreading area analysis were seeded with VSMCs at a concentration of 8500 cells/cm^2^. After 1, 3, and 7 days of cultivation, the samples were rinsed in phosphate-buffered saline (PBS, Sigma-Aldrich, Merck, Darmstadt, Germany), fixed with 4% paraformaldehyde for 15 min and permeabilized with 0.1% Triton X-100 in PBS. Subsequent staining with Hoechst #33258 (5 µg/mL; Sigma-Aldrich, Merck, Darmstadt, Germany) and with Phalloidin-TRITC (100 ng/mL; Sigma-Aldrich, Merck, Darmstadt, Germany) in PBS for 1 h at room temperature in the dark was used to visualize the cell nuclei (by Hoechst) and the cell membranes and the cytoskeleton (by Phalloidin).

The number of cells per cm^2^, the spreading area of the cells in µm^2^, and the morphology of cells were assessed from microphotographs taken by an epifluorescence microscope (Olympus IX-71 with a DP71 digital camera, Olympus Corp., Tokyo, Japan; objective 10×) or by an confocal laser scanning microscope (Leica TCS SP8, Leica, Germany; objective 40×) with the use of ImageJ FIJI image analysis software (version 1.52n; open-source [92]).

A resazurin assay was used to investigate the metabolic activity of the VSMCs, which is considered to be a marker of cell viability and proliferation. This assay is based on the activity of mitochondrial dehydrogenases in metabolically active cells, which convert blue-colored weakly fluorescent resazurin into pink-colored and highly fluorescent resorufin. The PS dishes were seeded with VSMCs in a density of 17,000 cells/cm^2^, and the PCL membranes were seeded with VSMCs in a density of 8500 cells/cm^2^. After 1, 3, and 7 days of cultivation, the PCL membranes in CellCrown inserts were gently transferred to new culture plates with fresh PBS. The PS dishes were also rinsed with fresh PBS. The mixture of the fresh culture medium supplemented with 10% FBS and resazurin at a final concentration of 40 mM (R7017; Sigma-Aldrich, Merck, Darmstadt, Germany) was incubated with the cells grown on the tested samples for 3 h in a humidified air atmosphere containing 5% CO_2_. The fluorescence of the medium/resazurin mixture was then measured (Ex/Em 530/590 nm) by a SynergyTM HT Multi-Mode Microplate reader (BioTek, Winooski, VT, USA). A solvent mixture taken from samples without cells was used as a blank control.

The potential immune activation of RAW 264.7 cells was evaluated by the amount of TNF-alpha and IL-1-beta molecules secreted into the culture medium by cells cultured on amine-PP-coated PCL NFs. Cell culture media were collected from the samples after 1 and 3 days of cultivation with RAW 264.7 cells. The media were subsequently centrifuged at 2000× *g* for 10 min to remove the cell debris. The concentrations of TNF-alpha and IL-1-beta in the culture supernatants were assessed by commercially available kits (Mouse TNF alpha ELISA Kit, Cat. No. ab229393 and Mouse IL-1 beta ELISA Kit, Cat. No. ab229440; both Abcam, Cambridge, UK). The fluorescence of the obtained mixture was measured (Ex/Em 530/590 nm) by a SynergyTM HT Multi-Mode Microplate reader (BioTek, Winooski, VT, USA). A solvent mixture without cells was used as a blank control, while a solvent mixture obtained from RAW 264.7 cells treated with bacterial lipopolysaccharide (LPS, Sigma-Aldrich, Merck, Darmstadt, Germany; 10 μg/mL incubated for 24 h before the culture media collection) was used as a positive control of TNF-alpha and IL-1-beta secretion.

### 4.6. Statistical Analysis

Thirty individual objects for each sample were measured by the SEM built-in tool to obtain the nanofiber and microparticle diameters. The WCA values were determined from ten different water droplets for each sample. A minimum of three individual biological samples for each experimental group and time interval, including the reference controls, were used in the biological experiments. At least 10 microphotographs of random areas were taken for each sample (a minimum of 30 images in total for each experimental group and for each time interval).

The results are presented in vertical dot plots, with each dot representing one biological sample of the replicates. All charts were made using OriginPro 8 (OriginLab Corporation, Northampton, MA, USA) or GraphPad Prism (GraphPad Software, San Diego, CA, USA). A statistical analysis of the acquired data was performed using SigmaStat 4.0 (Systat Software Inc., San Jose, CA, USA). Multiple comparison procedures were carried out by one-way ANOVA, followed by Student–Newman–Keuls test or by the Kruskal–Wallis one-way ANOVA on Ranks, Student–Newman–Keuls test for nonparametric data of cell metabolic activity on PP-coated PS samples. Values of *p* ≤ 0.05 were considered statistically significant for all experiments.

## 5. Conclusions

Amine plasma polymer films with various chemical structures, deposited on substrates with different morphologies, i.e., flat polystyrene culture dishes and nanofibrous PCL mats, were prepared from a CPA/Ar mixture via PECVD. Not only the deposition conditions, i.e., the average applied power, but also the type and the morphology of the substrate influenced the chemistry of amine-PPs. The nitrogen content and, therefore, the N/C ratio decreased with the rising applied power, and was noticeably lower on PCL NFs than on PS dishes. The material surface wettability altered according to the trend in the chemical structure of this surface. The hydrophilicity of the PP coatings dropped with a decreasing nitrogen content, and the amine-PP-coated PS dishes exhibited more hydrophilic behavior than the PCL NFs. All prepared amine-PP films, regardless of the discharge power that was applied, improved the initial adhesion of VSMCs to amine-PP-coated PS dishes and also to amine-PP-coated PCL nanofibers. Coatings deposited at the average RF power of 33 W (CPA-33) demonstrated the best combination of desirable functional properties: good water stability, a suitable amine group content, and favorable surface wettability and surface morphology. The CPA-33 PPs therefore induced the highest proliferation rate and the highest metabolic activity of VSMCs, regardless of the deposition substrate (PS dishes or PCL NFs). Although all nanostructured amine-PP-coated PCL NFs successfully decreased the proliferation rate of VSMCs, only CPA-33-coated PCL NFs promoted continuous proliferation of VSMCs during the 7-day-long cultivation. Importantly, the amine-PP-coated PCL NFs showed no signs of immunogenicity, as proved by TNF-alpha and IL-6 beta secretion by RAW 264.7 cells. These findings can be useful in a range of amine-PP film applications in medicine. Moreover, CPA-33-coated PCL NFs seem to be a promising material for the manufacture of vascular grafts, especially for grafts of small-diameter and with reconstructed *tunica media*.

## Figures and Tables

**Table 1:** Plasma deposition conditions.

*P*_av_ (W)	*P* (W)	D.C. (%)	PP Thin Film Reference in Text
10	30	33	CPA-10
33	100	33	CPA-33
150	150	100	CPA-150

## Supplementary Materials

Supplementary Materials can be found at https://www.mdpi.com/1422-0067/21/24/9467/s1.
