# Supplementary material for: Behaviour of Vascular Smooth Muscle Cells on Amine Plasma-Coated Materials with Various Chemical Structures and Morphologies
Source: Int J Mol Sci. 2020 Dec 12;21(24):9467. doi: 10.3390/ijms21249467 (PMC7763571; doi:10.3390/ijms21249467)
Supplement: Supplementary file 1 [file ijms-21-09467-s001.pdf]

International Journal of *Molecular Sciences*

MDPI

Supplementary Materials

Behaviour of Vascular Smooth Muscle Cells on Amine Plasma-Coated Materials with Various Chemical Structures and Morphologies

Ivana Nemcakova [1]*, Lucie Blahova [2] Petr Rysanek [3], Andreu Blanquer [1], Lucie Bacakova [1] and Lenka Zajíčková [2,4,5]

1 Laboratory of Biomaterials and Tissue Engineering, Institute of Physiology of the Czech Academy of Sciences, v.v.i., Videnska 1083, 142 20 Prague 4, Czech Republic; Andreu.BlanquerJerez@fgu.cas.cz (A.B.); lucie.bacakova@fgu.cas.cz (L.B.)

2 Central European Institute of Technology—CEITEC, Masaryk University, Kamenice 5, 625 00 Brno, Czech Republic; lucie.blahova@ceitec.muni.cz (L.B.); lenkaz@physics.muni.cz (L.Z.)

3 Department of Physics, Faculty of Science, University of J. E. Purkyne in Usti nad Labem, Pasteurova 15, 400 96 Usti nad Labem, Czech Republic; petr.rysanek@ujep.cz

4 Department of Physical Electronics, Faculty of Science, Masaryk University, Kotlarska 2, 61137 Brno, Czech Republic

5 Central European Institute of Technology—CEITEC, Brno University of Technology, Purkynova 123, 612 00 Brno, Czech Republic

* Correspondence: ivana.nemcakova@fgu.cas.cz

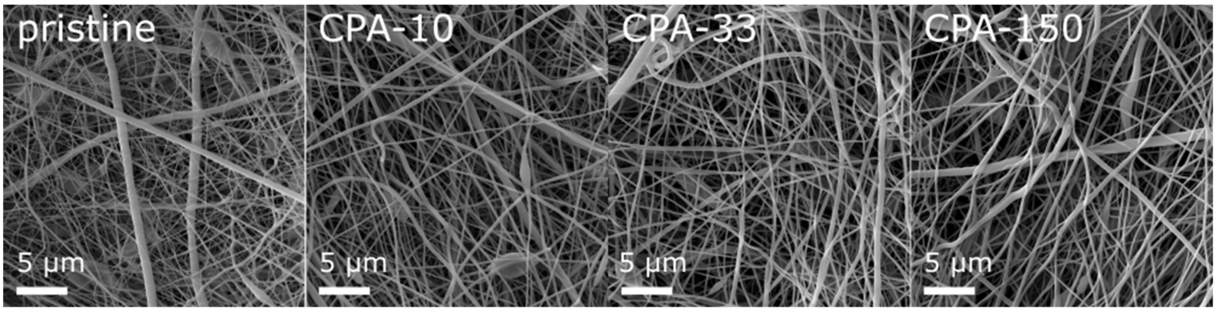

Figure S1. Micrographs of pristine and coated PCL NFs. CPA-10, CPA-33, CPA-150: PPs were deposited at the average RF power (P_{av}) of 10, 33, and 150 W, respectively.

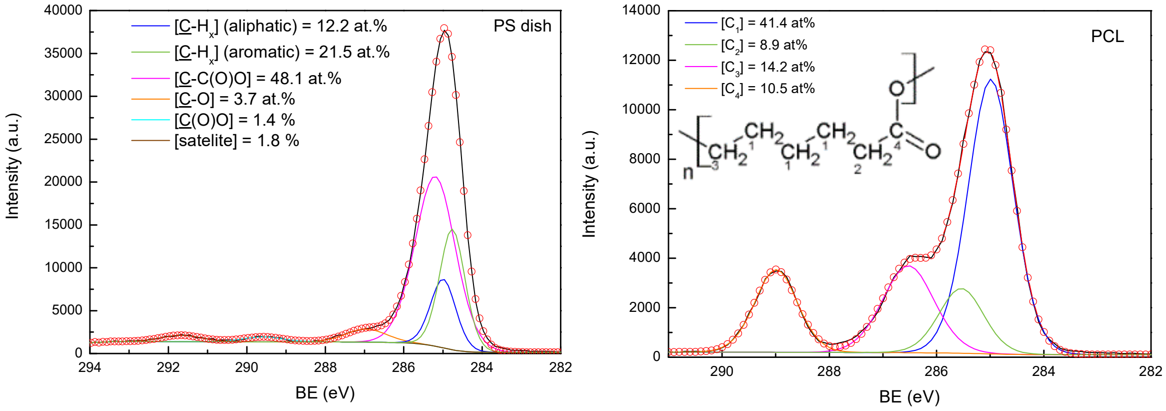

Figure S2. High-resolution XPS C1s spectra with identified functional groups for uncoated PS (left) and for uncoated PCL NFs (right).

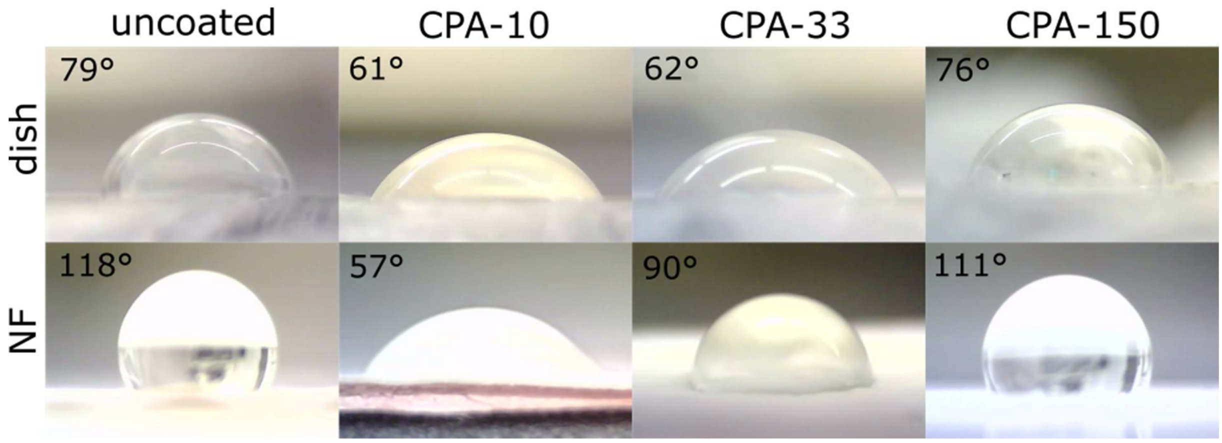

Figure S3. Shape of water droplets on uncoated and PP-coated PS dishes and PCL NFs aged two weeks with measured water contact angles. CPA-10, CPA-33, CPA-150: PPs were deposited at the average RF power (P_{av}) of 10, 33, and 150 W, respectively.

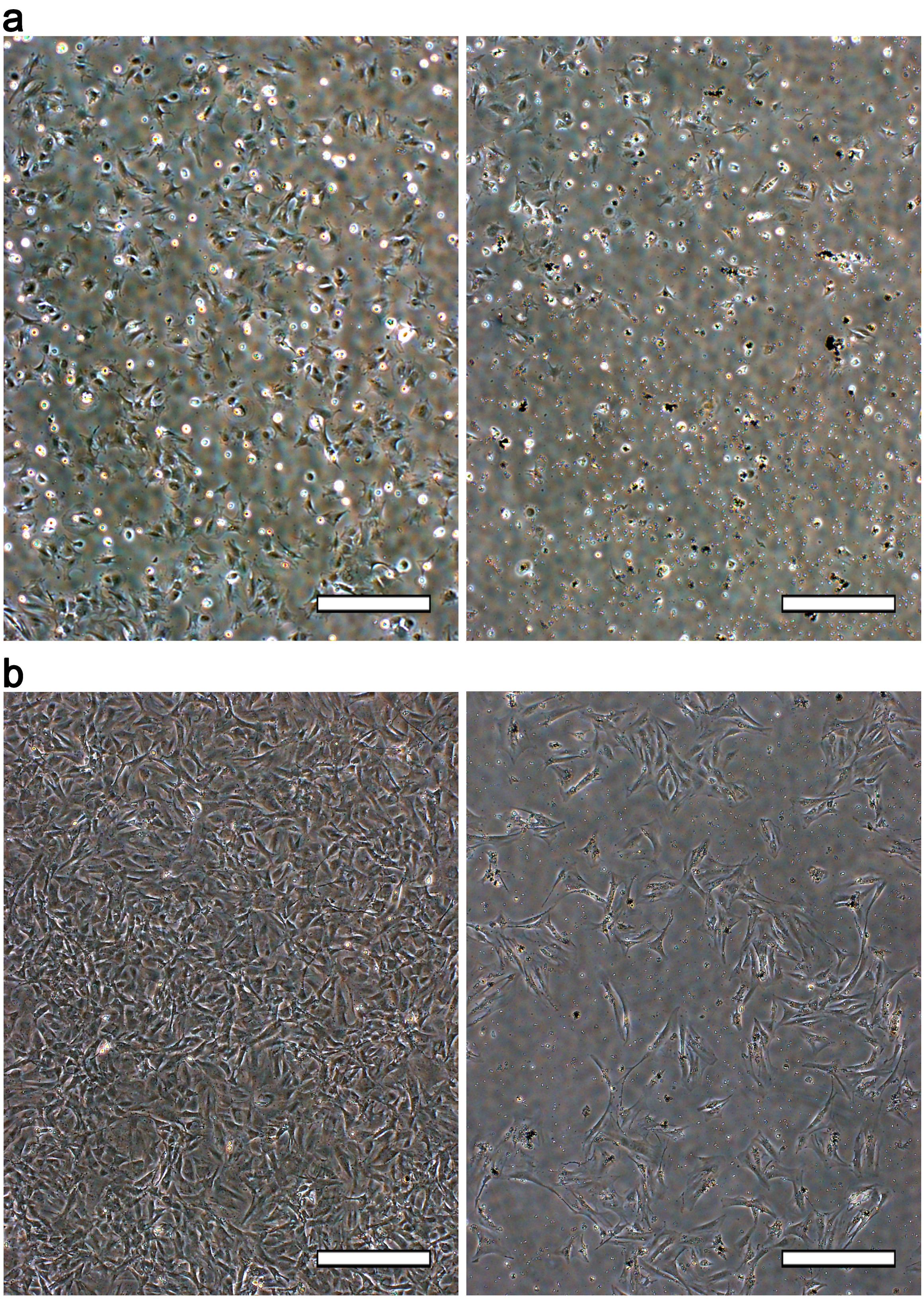

Figure S4. Morphology and population density of native VSMCs on CPA-150-coated PS dishes (i.e., PPs deposited at the average RF power (P_{av}) of 150 W) cultured (**a**) for 24 h and (**b**) for 7 days. (**a, b**) Images on the left represent major parts of the CPA-150 sample with lower amount of microparticles and higher cell densities with normal cellular morphology. Images on the right show less occurring parts of the CPA-150 sample (**a**) with mostly dead or unattached cells 24 h after seeding and (**b**) with cells with enlarged morphology after 7 days of culture. Microphotographs were taken by an epifluorescence Olympus IX-71 microscope. The scale bar depicts 400 μm.
